# A Multi-Endpoint Approach to Base Excision Repair Incision Activity Augmented by PARylation and DNA Damage Levels in Mice: Impact of Sex and Age

**DOI:** 10.3390/ijms21186600

**Published:** 2020-09-09

**Authors:** Nicola Winkelbeiner, Viktoria K. Wandt, Franziska Ebert, Kristina Lossow, Ezgi E. Bankoglu, Maximilian Martin, Aswin Mangerich, Helga Stopper, Julia Bornhorst, Anna P. Kipp, Tanja Schwerdtle

**Affiliations:** 1 Department of Food Chemistry, Institute of Nutritional Science, University of Potsdam, Arthur-Scheunert-Allee 114-116, 14558 Nuthetal, Germany; winkelbeiner@uni-potsdam.de (N.W.); vwandt@uni-potsdam.de (V.K.W.); fraebert@uni-potsdam.de (F.E.); martin@igzev.de (M.M.); 2 TraceAge—DFG Research Unit on Interactions of Essential Trace Elements in Healthy and Diseased Elderly (FOR 2558), 14558 Berlin-Potsdam-Jena-Wuppertal, Germany; kristina.lossow@uni-jena.de (K.L.); bornhorst@uni-wuppertal.de (J.B.); anna.kipp@uni-jena.de (A.P.K.); 3 Department of Molecular Nutritional Physiology, Institute of Nutritional Sciences, Friedrich Schiller University Jena, Dornburger Straße 24, 07743 Jena, Germany; 4 German Institute of Human Nutrition, Arthur-Scheunert-Allee 114-116, 14558 Nuthetal, Germany; 5 Institute of Pharmacology and Toxicology, University of Würzburg, Versbacher Straße 9, 97078 Würzburg, Germany; ezgi.bankoglu@uni-wuerzburg.de (E.E.B.); stopper@toxi.uni-wuerzburg.de (H.S.); 6 Department of Biology, Molecular Toxicology Group, University of Konstanz, Universitätsstraße 10, 78457 Konstanz, Germany; aswin.mangerich@uni-konstanz.de; 7 Food Chemistry, Faculty of Mathematics and Natural Sciences, University of Wuppertal, Gaußstraße 20, 42119 Wuppertal, Germany; 8 German Federal Institute for Risk Assessment (BfR), Max-Dohrn-Strasse 8-10, 10589 Berlin, Germany

**Keywords:** maintenance of genomic integrity, ageing, sex, DNA damage, base excision repair (incision activity), DNA damage response, poly(ADP-ribosyl)ation, liver

## Abstract

Investigation of processes that contribute to the maintenance of genomic stability is one crucial factor in the attempt to understand mechanisms that facilitate ageing. The DNA damage response (DDR) and DNA repair mechanisms are crucial to safeguard the integrity of DNA and to prevent accumulation of persistent DNA damage. Among them, base excision repair (BER) plays a decisive role. BER is the major repair pathway for small oxidative base modifications and apurinic/apyrimidinic (AP) sites. We established a highly sensitive non-radioactive assay to measure BER incision activity in murine liver samples. Incision activity can be assessed towards the three DNA lesions 8-oxo-2’-deoxyguanosine (8-oxodG), 5-hydroxy-2’-deoxyuracil (5-OHdU), and an AP site analogue. We applied the established assay to murine livers of adult and old mice of both sexes. Furthermore, poly(ADP-ribosyl)ation (PARylation) was assessed, which is an important determinant in DDR and BER. Additionally, DNA damage levels were measured to examine the overall damage levels. No impact of ageing on the investigated endpoints in liver tissue were found. However, animal sex seems to be a significant impact factor, as evident by sex-dependent alterations in all endpoints investigated. Moreover, our results revealed interrelationships between the investigated endpoints indicative for the synergetic mode of action of the cellular DNA integrity maintaining machinery.

## 1. Introduction

The limited chemical stability of DNA under physiological conditions facilitates the constant formation of a broad spectrum of DNA lesions [1]. Remaining unrepaired, DNA lesions can have deleterious consequences, that eventually can cause cancer and accelerate ageing [2]. Nuclear DNA damage accumulation and genomic instability seem to be important drivers in ageing, leading to cellular functional decline or loss of tissue homeostasis [3,4]. To maintain genomic stability, cellular mechanisms including the DNA damage response (DDR) and DNA repair are essential [2,5]. According to the general opinion stated in the literature, DNA damage increases with age, whereas the DNA repair capacity declines with age [5]. 

The major cellular repair pathway to remove the vast and frequently occurring spectrum of small oxidative base modifications, apurinic/apyrimidinic (AP) sites, and single-strand breaks is base excision repair (BER) (reviewed in [6,7,8,9,10]). 

There are different approaches to study BER processes. The predominant approach includes the preparation of a cell or tissue extract that contains the active repair enzymes and is incubated with DNA damage-containing substrates. These can be radioactively or fluorescently labelled oligonucleotides or plasmids with an incorporated DNA lesion, and, in case of comet assay-based repair assays, damaged nucleoid DNA [11,12,13,14,15]. Many extract-based assays monitor specific BER initiating incision events performed by DNA glycosylases [11,12,15]. Also, all further enzymatic steps of BER, such as activities of polymerases and ligases, can be assessed [13,14,15]. Assays yielding information on the repair process in its entirety are considered as the most comprehensive approach to study BER. The same is true for assays based on host cell reactivation [16,17]. However, the application of these assays to tissue samples is still more limited because tissue is a more complex matrix than cultured cells and, in some cases, intact vital cells are required for assay performance.

**Figure 1:**
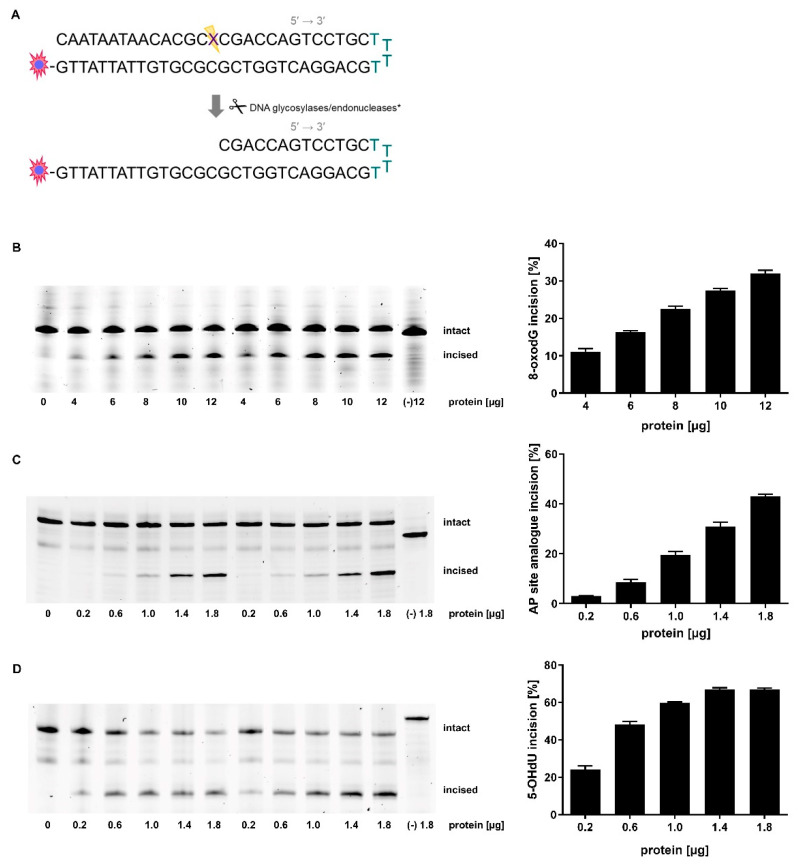
Incision activity assay. Schematically shown is the 3’ Cy5-labelled oligonucleotide base sequence of the 8-oxodG/ AP site analogue containing oligonucleotide in hairpin structure (5-OHdU containing oligonucleotide slightly differing) before and after lesion incision (**A**). Major relevant DNA glycosylases/endonucleases (*) for lesion (X) incision: X = 8-oxodG → OGG1 (+ APE1), X = AP site analogue → APE1, X = 5-OHdU → NEIL1/2 (+ polynucleotide kinase) / NTH1 (+ APE1). Incision activity of different amounts of tissue extract protein was assessed towards (**B**) the 8-oxodG, (**C**) the AP site analogue, and (**D**) the 5-OHdU containing oligonucleotide by fluorescence detection (full-length gels are shown in Figure S3). Shown are polyacrylamide gels (left, 2 replicates of each applied protein amount are shown) and mean values + SEM (*n* = 4 (technical replicates of one animal)) of corresponding quantified BER incision activity (right). Quantification was performed by analysis of percentage of incised (lower band) versus intact (upper band) oligonucleotide for each lane. As negative control for each oligonucleotide with a DNA lesion, a damage-free oligonucleotide with the respective base sequence and the correct pairing base (G instead of 8-oxodG and the AP site analogue; C instead of 5-OHdU; marked as (-)) was designed and incubated according to the assay protocol with tissue extract protein.

Within this work, we established a highly sensitive non-radioactive BER incision activity assay to quantify the efficiency of the BER damage incision step in murine liver tissue. The assay’s advantage is that BER incision activity can be determined from one single tissue extract for three DNA lesions. To achieve this, we applied oligonucleotides that contain the oxidative base modifications 8-oxodG, 5-OHdU, or an AP site analogue (schematically shown in Figure 1A). 8-oxodG is numerously formed (100–500 times per day in a mammalian cell) because guanine has the lowest oxidation potential among the nucleobases and is therefore particularly vulnerable to reactive oxygen species (ROS) attack [1,18]. Repair of 8-oxodG in mammals is predominantly initiated by the 8-oxoguanine DNA glycosylase 1 (OGG1) [12,19,20]. 5-OHdU is formed by oxidative deamination of cytosine, mostly driven by ROS [21]. It is mainly removed by the nei-like DNA glycosylases 1 and 2 (NEIL1/2), and the nth-like DNA glycosylase 1 (NTH1) [22,23,24,25]. The third DNA lesion investigated is an AP site, which is a nucleoside in DNA that has neither a purine nor a pyrimidine base. It can be formed by spontaneous hydrolysis under physiological conditions (approximately 10,000 times per day per human genome) or in the process of BER [1,26]. AP endonuclease 1 (APE1) is the major mammalian enzyme responsible for processing AP sites [27]. All of these DNA lesions are premutagenic [28,29]. If they remain unrepaired, a variety of cellular problems result, and eventually persistent DNA damage can be fixed in mutations, thus contributing to genomic instability [26]. The possibility to analyse the three above-described BER incision events in murine tissue presents a powerful tool to investigate cellular capacity to recognize specific DNA lesions and initiate DNA repair. 

Next to DNA repair, the DDR fulfils a crucial function within the cellular response to incurred DNA damage by mediating the initialization of repair processes and recruiting the respective repair enzymes. Outstanding in its versatile physiological functions, among others in the DDR and BER, is the multi-functional enzyme family of poly(adenosine diphosphate (ADP)-ribose) polymerases (PARPs). PARPs serve as damage sensor, initiate and support BER, and facilitate DNA accessibility to the respective repair enzymes [30]. The enzymes catalyse the transient post-translational modification of proteins with poly(ADP-ribose) (PAR) in response to genotoxic insults such as DNA strand breaks, a process referred to as PARylation. During PARylation, linear or branched ADP-ribose chains of up to 200 ADP-ribose units are formed that affect acceptor protein function due to the negative charge of the ADP-ribose polymer [31]. As fast as PAR is formed in response to DNA damage, as rapidly it is degraded by the poly(ADP-ribose) glucohydrolase and the PAR hydrolase 3 [32,33]. In vitro studies showed that PARylation promotes recovery from genotoxic stress [34]. While efficient PARylation is thought to delay ageing, PARP1 deficiency has been shown to facilitate ageing [35,36]. To assess the tissue PARylation status, we quantified cellular PAR in the mouse livers via stable isotope dilution liquid chromatography tandem mass spectrometry (LC-MS/MS) [37].

Further, the analysis of DNA damage levels can give an indication on the integrity of the cellular DNA and is directly related to the fidelity of DDR and DNA repair. DNA damage imbalances tissue homeostasis and is discussed as a central cause and consequence of ageing and age-related disease (reviewed in [3]). Among the highly diverse myriad of DNA damage types associated with ageing, including DNA strand breaks, cross-links, and (oxidative) base modifications, DNA single- and double-strand breaks, as well as AP sites can for example be assessed by alkaline comet assay [2,5,38]. This single cell gel electrophoresis is a standard method frequently used in genotoxicity testing and biomonitoring, convincing by its sensitivity, simplicity, and reproducibility [38,39]. Compared to other methods used to quantify DNA damage, for example via LC/MS-MS, only a small amount of tissue is needed for assay performance and obtained results are on the level of individual cells [38]. Yet, via highly sensitive LC-MS/MS analysis, also specific complex DNA lesions, relevant to ageing, can be targeted, which are not accounted for by comet assay analysis [40].

Innovative and feasible methods to analyse the efficiency of the cellular response to DNA damage represent valuable tools in basic research and in the assessment of potential benefits and risks, for example in development and targeting of drugs. By the combined assessment of BER incision activity, the PARylation status, and DNA damage levels in the liver of adult and old mice of both sexes, this paper aims to contribute to a deeper understanding of the impact of age and sex on the genomic stability maintaining machinery.

Within this study, reliable methods for the analysis of the cellular DDR and DNA repair status in murine liver were established and validated. Implementation of the described methods revealed no significant impact of ageing on the assessed endpoints. Yet, animal sex evidently affected BER incision activity, PARylation, and DNA damage levels. Further, our results revealed predictive relationships between DNA damage level and PARylation as well as between DNA damage level and BER incision activity. 

## 2. Results

### 2.1. Adaption of Assay Parameters

To prepare a suitable tissue extract for the incision assay, we adapted different parameters of the cell-based protocol [12]. We added the non-ionic surfactant Triton X-100, known to improve cellular membrane permeability [41], in a final concentration of 1% to the extraction buffer. This enhances the release of the soluble protein fraction from the tissue-bound liver cells that have a more complex matrix than cultured cells. Besides the extraction buffer from the cell-based protocol, we tested a 4-(2-hydroxyethyl)piperazine-1-ethanesulfonic acid (HEPES)-based extraction buffer, which was reported to be efficient in a comet-based DNA repair assay [42]. Eventually, incision activity was reduced applying the HEPES-based extraction buffer (Figure S1A, buffer B). Thus, we chose the extraction buffer from the cell-based incision assay (Figure S1A, buffer A). Assays to determine DNA repair activity in extracts from rodent tissues have been reported to be susceptible to disturbance by non-specific nuclease activity [42,43,44]. Langie et al. showed that the addition of 1.5 µM aphidicolin (APC) to tissue extracts could reduce non-specific nuclease activity, while DNA repair incision activity could be improved in a comet-based in vitro repair assay [42]. Therefore, we included 1.5 µM APC in our extraction buffer. Homogenization of the liver tissue was performed in the optimized extraction buffer using a bead ruptor and zirconia beads. We added 1 mL of extraction buffer to 50 mg of frozen liver tissue to obtain optimal protein concentrations (7–10 mg/mL). This ensured best storage stability and handling of the tissue extract in the assay procedure. Interestingly, we found that the centrifugation step with 10 kDa filter units previously used for cell extracts is not needed for murine liver extracts (Figure S1B). Finally, we quantified fluorescence of the fluorophore with high sensitivity in the fmol range, applying a charge-coupled device (CCD)-camera based imaging system with LEDs as light source. 

### 2.2. Optimal Protein Amounts, Storage Conditions, and Tissue Processing

According to the adapted protocol, tissue extracts from murine liver were prepared and the optimal protein amount was determined for each of the three damage-containing oligonucleotides. In this case, the optimal protein amount is characterized by the degree of incision activity. It should allow to detect an increase as well as a decrease in incision activity to make the assay capable of showing changes in incision activity in both directions. Based on the results for the 8-oxodG containing oligonucleotide, 8 µg tissue extract protein were chosen, whereas for the AP site analogue and 5-OHdU containing oligonucleotides, 1 µg of tissue extract protein appeared to be most suitable. Applying these protein amounts resulted in incision activities within a linear range, therefore meeting the assay criteria described above. Differences in incision activity levels refer to enzyme activities under assay conditions. To ensure quantification of only specific incision events, we incubated damage-free oligonucleotides with the respective base sequences, but with an intact pairing base with tissue extract. The control samples showed that no unspecific incision occurred and that the oligonucleotides were stable under assay conditions (Figure 1B–D). 

**Figure 2:**
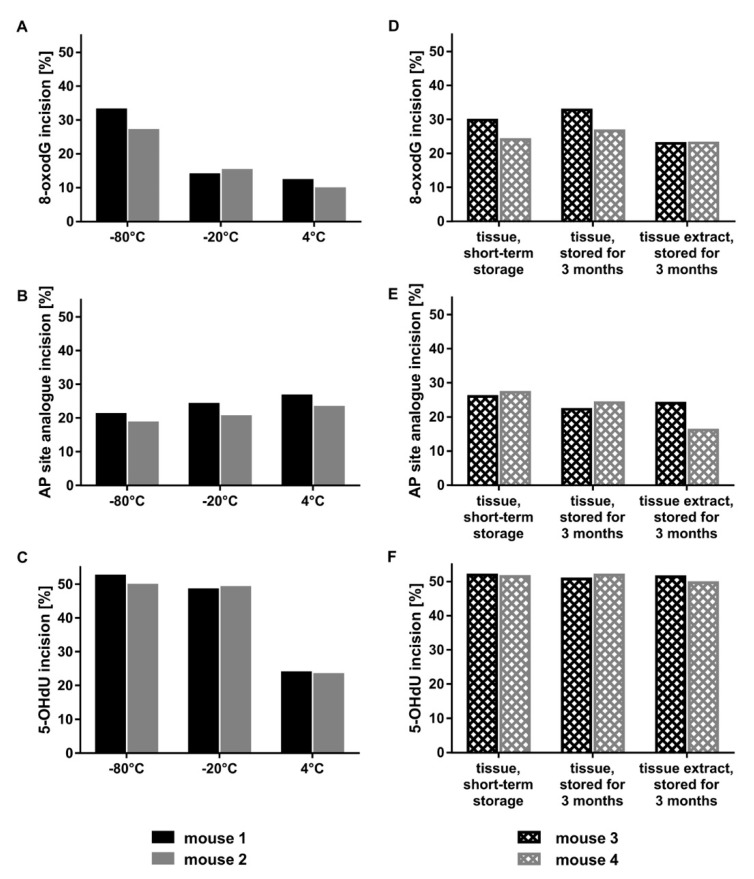
Storage stability. For assessment of short-term storage stability of murine liver extracts, incision activity was determined towards (**A**) the 8-oxodG, (**B**) the AP site analogue, and (**C**) the 5-OHdU containing oligonucleotide after 5-day storage of liver tissue extracts at −80, −20, or 4 °C. Tissue extract protein amounts of two different mice were applied as follows: 6 µg for the 8-oxodG, 1 µg for the AP site analogue and 5-OHdU containing oligonucleotide. Regarding long-term storage stability, incision activity was determined towards the (**D**) 8-oxodG, (**E**) AP site analogue, and (**F**) 5-OHdU containing oligonucleotide after short-term (max. 5 days) or long-term storage (3 months) of liver tissue or after long-term storage (3 months) of tissue extracts. Tissue extract protein amounts of two different mice were applied as follows: 8 µg for 8-oxodG, 1 µg for the AP site analogue and 5-OHdU.

Incision activity of tissue extracts was not affected by storage at −80 °C for 24 h or five days. Nevertheless, storing extracts at 4 °C or −20 °C resulted in a dramatic decrease of incision activity, especially after five-day storage at 4 °C (Figure 2A–C). Additionally, incision activity was compared with regard to long-term storage. Hence, liver tissue extracts of two different mice were prepared from liver that was stored at −80 °C only for a few days or three months. Incision activity of the freshly prepared tissue extracts was compared to incision activity of the three months at −80 °C stored tissue extracts and to incision activity of tissue extracts prepared from the three months at −80 °C stored liver tissue (Figure 2D–F). Examination of incision activity revealed that the intact liver tissue and the prepared tissue extracts can be stored for three months at −80 °C without remarkably affecting incision activity. Furthermore, we tested if tissue treatment prior to preparation of tissue extracts impacts on incision activity. Thus, a small piece of tissue was either frozen in liquid nitrogen (directly after harvesting) and kept at −80 °C until preparation of tissue extract, or it was frozen by squeezing it between two liquid nitrogen cooled stainless steel plates. The squeezed liver tissue was further used in this form for preparation of tissue extract or was additionally homogenized under liquid nitrogen cooling with mortar and pestle. Best results were obtained by preparing tissue extracts from tissue directly frozen after harvesting without any tissue treatment (data not shown).

### 2.3. Application of the Incision Activity Assay to a Cohort of Adult and Old Mice

**Figure 3:**
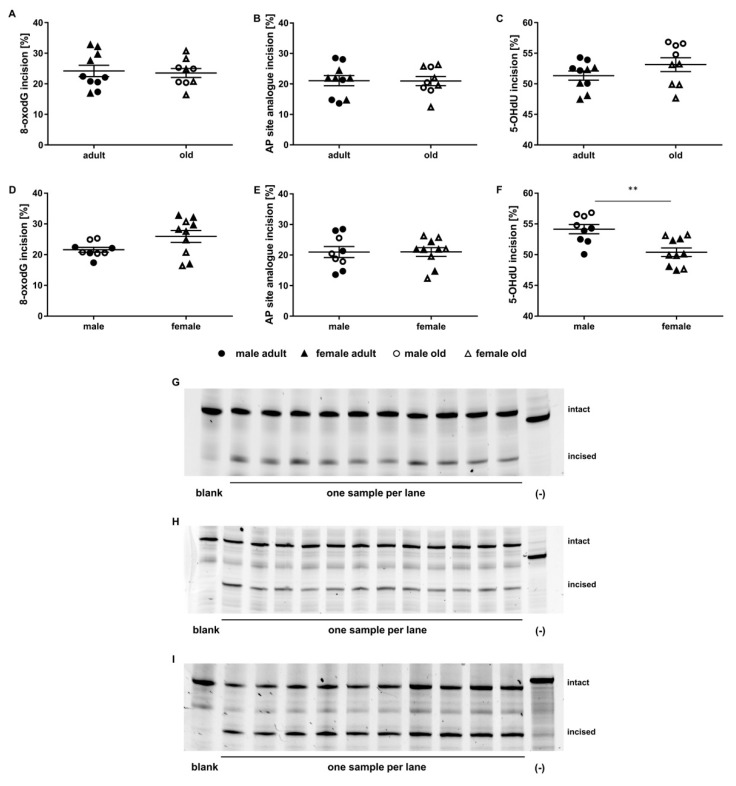
Impact of age and sex on incision activity. Shown is BER incision activity (individual single measurement scores per animal and group mean ± SEM) of murine liver of adult (24 weeks, *n* = 10) and old (109-114 weeks, *n* = 9) animals of both sexes (female: *n* = 10, male: *n* = 9) towards (**A**,**D**) the 8-oxodG, (**B**,**E**) the AP site analogue, and (**C**,**F**) the 5-OHdU containing oligonucleotide. Additionally, one representative polyacrylamide gel is shown for (**G**) the 8-oxodG, (**H**) the AP site analogue, and (**I**) the 5-OHdU containing oligonucleotide (full-length gels shown in Figure S3). The left lane shows the respective oligonucleotide blank followed by single sample measurements per lane and a negative control (as described above, marked as (-)) in the right lane. t-test, ** *p* ≤ 0.01.

**Figure A1:**
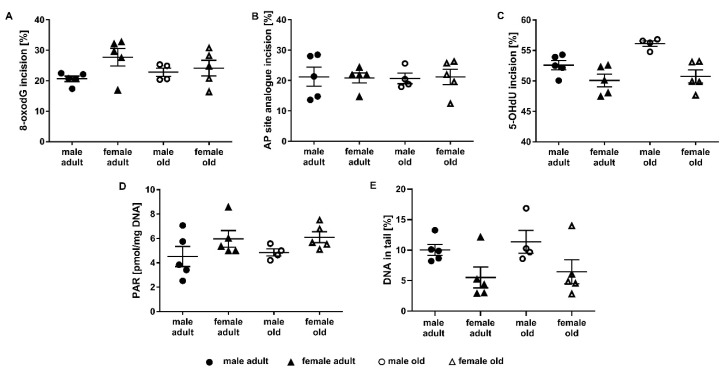
Impact of age and sex on incision activity, PARylation, and DNA damage levels. Shown is BER incision activity (individual single measurement scores per animal and group mean ± SEM) of murine liver of adult (24 weeks, *n* = 10) and old (109-114 weeks, *n* = 9) animals of both sexes (female: *n* = 10, male: *n* = 9) towards (**A**) the 8-oxodG, (**B**) the AP site analogue, and (**C**) the 5-OHdU containing oligonucleotide. Additionally, shown are PARylation levels (individual single measurement scores per animal and group mean ± SEM) (**D**) and DNA damage levels (individual single measurement scores (two technical replicates) and group mean ± SEM) (**E**).

The established incision activity assay was applied to study age and sex effects on incision activity in a group of 19 mice of both sexes that were fed with a standard chow and were either 24 weeks (adult, *n* = 10), or 109–114 weeks old (old, *n* = 9). Incision activity towards the 8-oxodG containing oligonucleotide was mainly between 20 and 30% for most of the animals (Figure 3A). Mean incision activity within the groups of adult (24.21 ± 1.85% incision) and old animals (23.55 ± 1.48% incision) showed no significant differences. Incision activity regarding the AP site analogue containing oligonucleotide was 20% for the majority of animals, with some animals showing increased or decreased incision activities in both age groups (Figure 3B). As for the 8-oxodG containing oligonucleotide, we did not observe age-related differences in the mean incision activity (adult mice: 21.05 ± 5.29% incision; old mice: 20.94 ± 1.51% incision). Mean incision activity towards the 5-OHdU containing oligonucleotide was 50–60% incision on average with a tendency to be higher in old animals (53.12 ± 3.35% incision) than in adults (51.33 ± 2.34% incision) (Figure 3C). Interestingly, for 5-OHdU we observed a significant sex-specific difference in mean incision activity that did not manifest for the 8-oxodG or AP site analogue containing oligonucleotides (Figure 3D–F). Male animals showed a significantly higher mean incision activity towards the 5-OHdU containing oligonucleotide than females (male: 54.15 ± 2.26% incision; female: 50.40 ± 2.27% incision). In Figure A1A–C, results are shown differentiated for sex and age of the mice at the same time. Representative gels of the sample measurements for each of the three oligonucleotides are shown in Figure 3G–I. 

### 2.4. Determination of Parylation Levels in Murine Liver

**Figure 4:**
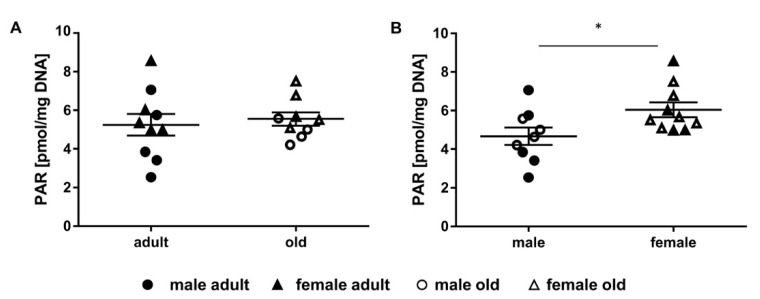
Impact of age and sex on PARylation. Shown are PARylation levels (individual single measurement scores per animal and group mean ± SEM) of murine liver of adult (24 weeks, *n* = 10) and old (109–114 weeks, *n* = 9) animals of both sexes (female: *n* = 10, male: *n* = 9). (**A**) adult versus old, (**B**) male versus female animals. t-test, * *p* ≤ 0.05.

Mean PARylation levels in the livers of the studied animals were in general between 2 and 8 pmol PAR/mg DNA and did not differ strongly between adult (5.25 ± 0.56 pmol PAR/mg DNA) and old mice (5.55 ± 0.34 pmol PAR/mg DNA) (Figure 4A). Remarkably, PARylation levels within the group of adult animals showed a higher variation compared to the more consistent PARylation levels within the group of the old animals. Though, sex-specific differences became apparent for the PARylation levels. Female mice showed significantly higher mean PARylation levels (6.04 ± 0.38 pmol PAR/mg DNA) than male animals (4.67 ± 0.45 pmol PAR/mg DNA) (Figure 4B). In Figure A1D, results are shown differentiated for sex and age of the mice at the same time.

### 2.5. Assessment of DNA Damage in Murine Liver Samples 

**Figure 5:**
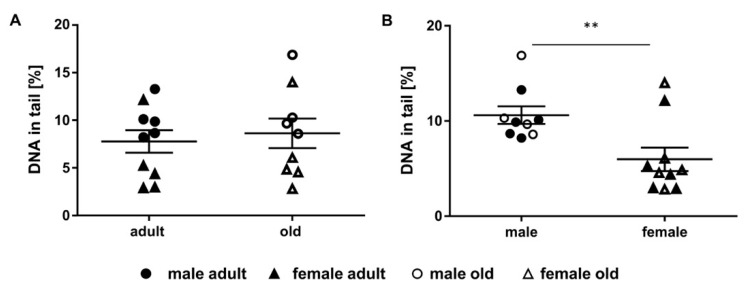
Impact of age and sex on DNA damage levels. Shown is DNA in tail (individual single measurement scores (two technical replicates) and group mean ± SEM) of murine liver of adult (24 weeks, *n* = 10) and old (109-114 weeks, *n* = 9) animals of both sexes (female: *n* = 10, male: *n* = 9). (**A**) adult versus old, (**B**) male versus female animals. t-test, ** *p* ≤ 0.01.

The mouse livers showed no significant differences in mean DNA damage level between the groups of adult (7.77 ± 1.18% DNA in tail) and old animals (8.63 ± 1.55% DNA in tail) (Figure 5A). Yet, we found a significant difference in DNA damage between males and females (Figure 5B). Male animals (10.61 ± 0.93% DNA in tail) showed significantly increased mean levels in DNA damage compared to female animals (5.98 ± 1.24% DNA in tail). In Figure A1E, results are shown differentiated for sex and age of the mice at the same time. 

### 2.6. Correlation of Genomic Stability Endpoints 

**Figure 6:**
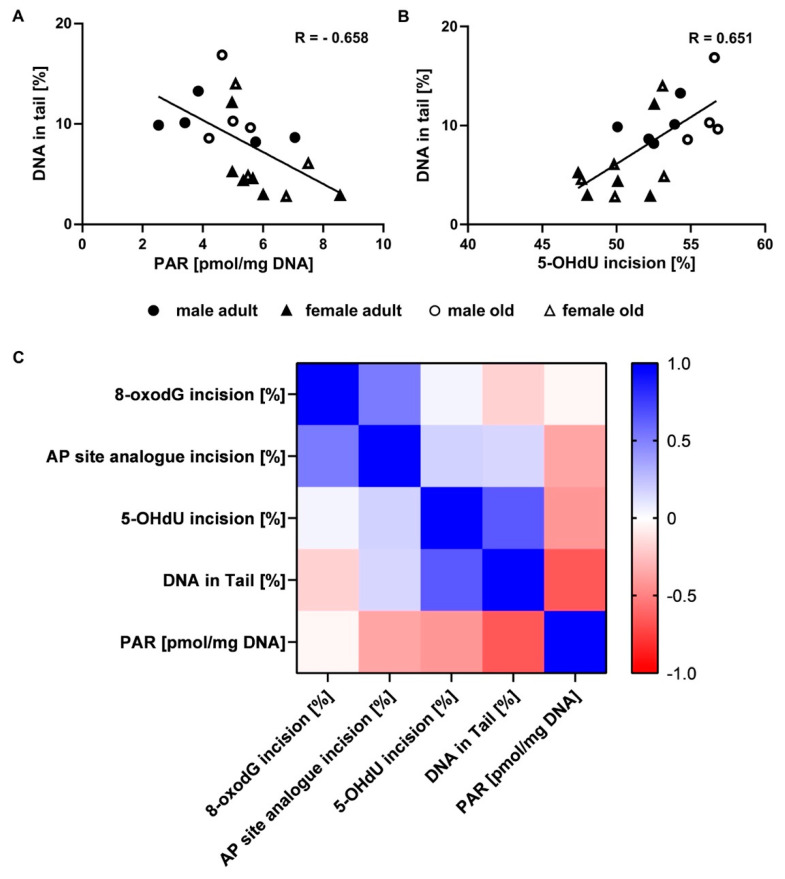
Relationships between genomic stability endpoints analysed by explorative correlation analysis. DNA damage and PARylation levels showed an inverse relationship (**A**) (*p* = 0.002, Spearman’s R = -0.658), while DNA damage levels and incision activity towards the 5-OHdU containing oligonucleotide were positively related (**B**) (*p* = 0.003, Spearman’s R = 0.651). The black line represents the linear regression. The correlation matrix-based heat map indicates Spearman’s correlation coefficient R between the analysed genomic stability endpoints (**C**).

DNA damage and PARylation levels were inversely related (*p* = 0.002, Spearman’s R = −0.658; Figure 6A), while DNA damage levels and 5-OHdU incision activity were positively correlated (*p* = 0.003, Spearman’s R = 0.651; Figure 6B). A correlation matrix-based heat map including all results is shown in Figure 6C.

## 3. Discussion

Within this study, we present a fluorescence-based, highly sensitive, reproducible, multi-endpoint incision activity assay to determine BER incision activity in tissue extracts from murine liver. The established protocol allows for simultaneously assessing incision activity towards three common DNA lesions proceeding from one single tissue extract, thereby saving time, effort, and animal derived tissue. In an ageing mouse cohort including animals of both sexes, we analysed BER incision activity, PARylation, and DNA damage levels in the liver. We have not performed analysis of specific tissue protein levels or quantity of the analysed DNA repair proteins, as it has been shown before that there is no relationship between the activity of DNA repair enzymes and mRNA or protein levels, respectively [45].

Our results for the incision activities towards different DNA lesions showed no significant age-related changes (Figure 3A–C). On the contrary, incision activities remained quite stable with increasing age. In the literature, this is controversially discussed. In rodent tissues, BER capacity has been reported to decrease [46,47] or increase [48], while others found a steady-state repair capacity [49]. In line with the latter, we found stable incision activities for both 8-oxodG, the AP site analogue, and 5-OHdU containing oligonucleotides independent of the age of the mice. In another study, for 8-oxodG, a significant age-related increase in damage levels (by 47% in liver) was observed in liver of old (24–26 months) compared to young (6 months) C57BL/6 mice. The authors also investigated the capacity of tissues from young and old mice to remove 8-oxodG after low-dose γ-irradiation. Interestingly, removal capacity for 8-oxodG did not strongly differ between young and old C57BL/6 mice, indicating a consistent DNA repair capacity with increasing age [49]. Further, Souza-Pinto and colleagues found a significant increase in mitochondrial repair capacity with increasing age, but nuclear incision activity was not affected [50]. In another study, APE1 protein and mRNA levels along with enzyme activity in total tissue extracts of mouse liver did not differ significantly between young (4 months), middle-aged (10 months), and old (20 months) mice. However, age-related differences in localisations of APE1 between nucleus, cytoplasm, and mitochondria were observed [51]. With the incision assay presented in this work, it is not possible to distinguish between nuclear or mitochondrial DNA repair capacity. Thus, we can assess overall capacity of the tissue to initialize BER and remove the respectively studied DNA lesions, which is, considering our results, not affected by age-related changes. In contrast, Gorniak and colleagues reported a two-fold increase in incision activity in livers from old (32 months) C57BL/6 mice compared to young mice (6 months) determined via comet-based incision assay [48]. This approach also targets the initial step of BER by using the photosensitiser Ro 19-8022 and visible light-damaged substrate cells that mainly contain 8-oxodG. It is surprising that the results the authors found are so contrary from what we observed. Besides the different attempts to measure BER incision activity, the studied mice, their age, and the tissue investigated were comparable. However, 5-OHdU incision activity also shows the tendency to increase with increasing age of the animals. This indicates a higher activity of the respective DNA glycosylases in old animals compared to the adult ones.

Besides BER incision activity, we quantified PARylation levels in the livers of the mice (Figure 4A). Although DNA repair is commonly studied in murine liver, PARylation levels have been investigated in a limited number of studies so far. PARylation levels showed no significant differences regarding the age of the animals. An important aspect to consider in this context is the dynamic interplay between BER coordinating enzymes and the redundancy of the BER system. The BER protein-protein interactions such as between OGG1 and PARP1 [52] as well as NEIL1 and PARP1 [53] impact on the enzymes’ activities in multiple ways. Thus, incision activities towards 8-oxodG (mainly OGG1 activity) and towards 5-OHdU (mainly NTH1 and NEIL1/2 activity) as well as PARylation levels that show no age-related changes for each, could be interpreted as key players in a well-regulated overall BER system that is not affected by the animals’ age. In line with this, damage levels assessed by alkaline comet assay were not affected by the age of the animals (Figure 5A). In general, it is assumed that the frequency of DNA damage increases in ageing [4]. Yet, various studies in humans showed contradictory results [54] that argue against the theory of DNA damage accumulation in ageing. Surprisingly, data evidently proving ageing as causally correlated with increasing DNA damage levels is scarce. In a mouse study with normally and prematurely aged mice, DNA damage was quantified by measuring inhibition of long-distance polymerase chain reaction. In liver tissue of inbred C57BL/6 mice (young = 4 months old, old = 28 months old) only a marginal increase in DNA damage was observed with increasing age. Surprisingly, DNA repair defective mice (ERCC excision repair 1^-/Δ^ and Ku80 mutant mice, young = 5 months old, old = 12 months old) showed no increase in DNA damage in the liver with increasing age. Consequently, the authors conclude that neither normal nor premature ageing was associated with an increase in DNA damage [55]. These results rather support our findings of no increase in DNA damage in liver with ageing than the theory of DNA damage accumulation in ageing. This might indicate other cellular factors contributing to ageing. Nevertheless, by alkaline comet assay only single- and double-strand breaks, as well as alkali-labile sites are quantified [38]. Other ageing-associated DNA damage, such as DNA crosslinks and (oxidative) base modifications, are not taken into account [2,5]. In this context, Izzotti et al. revealed increased 8-oxodG as well as DNA crosslink levels associated with ageing in several mouse organs using highly sensitive LC-MS/MS techniques [56]. Hence, to avoid underestimation of DNA damage levels and to get a better understanding of the different occurring DNA damage types related to ageing, further studies are needed, including sensitive techniques to quantify several DNA damage types, such as LC-MS/MS-techniques [40].

To draw conclusions from the analysed endpoints, it is crucial to consider results holistically. Thus, exploratory correlation analysis revealed interesting interrelationships. We found a negative relationship between DNA damage and PARylation levels (Figure 6A). This seems contradictory to the observation that PARylation transiently increases in response to genotoxic insults and DNA damage [33,34]. Based on this, one would expect an increased PARylation in association with high damage levels, and so, a positive correlation. Yet, we cannot exclude that the overall damage level, including damage that was not assessed by alkaline comet assay, might be different from our results and hence the observed correlation might be distorted. However, it was also shown that PARylation of histones and the respective damage sites results in chromatin remodelling and relaxation of the chromatin structure. Resulting from this, accessibility of the damage site is improved and binding of DNA repair factors enhanced upon PARylation [33,57]. Thus, the negative relationship we observed could also reflect a repair facilitating effect of PARylation concomitant with decreasing DNA damage levels. In line with both discussed options, female mice showed moderately higher PARylation and distinctly lower DNA damage levels than male animals (Figure 4B and Figure 5B). This could possibly be explained by considering that a significant increase in PARP1 activity in vitro was shown with factor ten increasing estradiol concentrations [58]. In general, female mice have estradiol concentrations ten times higher than males [59]. Thus, the observed sex-specific differences could be interpreted to follow the inverse relationship between PARylation and DNA damage levels.

Further, DNA damage levels were positively associated with 5-OHdU incision activity (Figure 6B). This indicates a causal relationship between low DNA damage levels and reduced activity of NEIL 1/2 and NTH1. Vice versa, increased DNA damage levels appear to enhance repair enzyme activity. Enhanced DNA repair enzyme activity at the same time results in an increased number of BER intermediates, in particular single-strand breaks. These further increase DNA damage levels as assessed by alkaline comet assay. In line with these findings, female mice showed lower DNA damage levels and lower 5-OHdU incision activity compared to the males (Figure 3F and Figure 5B).

Taken together, our results suggest that genomic stability maintaining and DNA damage reducing mechanisms in this study functioned more efficiently in female than in male mice. 

However, our results also indicate that BER initiating enzyme activity might be affected by the DNA damage level and monitors the existing amount of DNA damage. Thus, quantification of the specific DNA lesions that are subject to the respective repair enzymes would be of interest. Moreover, monitoring of further enzymatic steps, such as gap-filling and termini clean-up by polymerases, in particular polymerase β and the BER completing strand ligation step by ligases I and IIIα [60], would be required for additional validity regarding interpretation of results. Especially as the polymerase-mediated termini clean-up is indicated as the rate limiting step of BER [61], assessment of polymerase efficiency would add valuable information to this experimental setting. If by high fidelity of DNA glycosylases and APE1, many intermediate strand-breaks are generated, reduced polymerase activity could also result in strand-break accumulation and increased DNA damage levels as assessed by comet-assay analysis. Thus, aging could also affect enzymatic steps of BER aside from damage recognition and incision. Hence, an effect of aging on BER in this study cannot be excluded by the analysed endpoints.

Our results showed how the complex cellular mechanisms of DDR and DNA repair impact on each other, manifesting for example in an altered DNA damage status. Analysis of BER incision activity, PARylation status, and DNA damage levels can only give insights into the functionality of the cellular machinery maintaining genomic integrity. Yet, analysis of these endpoints can contribute to a deeper understanding of impacting factors such as sex and age on the cellular DNA damage defending mechanisms. 

## 4. Materials and Methods 

### 4.1. Animal Husbandry

Animal experiments were approved by and conducted following national guidelines of the Ministry of Environment, Health and Consumer Protection of the federal state of Brandenburg, Germany (2347-44-2017) and institutional guidelines of the German Institute of Human Nutrition Potsdam-Rehbruecke, Germany. Mice were housed in polycarbonate cages and kept under diurnal 12 h light/dark cycles with light beginning at 6:30 AM; room temperature and humidity was kept constant at 22 °C and 55%, respectively. Food (V1534, Ssniff, Soest, Germany) and water were offered *ad libitum*. 

Tissues were obtained from C57BL/6JRj mice of both sexes at an age of 24 (young adult, referred to as adult, 6 months) or 109–114 weeks (old adult, referred to as old, 28 months) anesthetised by isoflurane (Cp-pharma, Burgdorf, Germany), after blood withdrawal by heart puncture. Fresh liver tissue was cut in aliquots, snap-frozen in liquid nitrogen, and stored at −80 °C until further use. All analyses were conducted with samples that were fully blinded starting from the point of tissue harvesting.

### 4.2. Materials

2-Amino-2-(hydroxymethyl)-1,3-propanediol (Tris), Tris hydrochloric acid (HCl), sodium chloride (NaCl), sodium hydroxide (NaOH), disodium ethylenediaminetetraacetate (Na_2_EDTA), acrylamide solution, urea, boric acid, N,N,N′,N′-tetramethylethylenediamine (TEMED), ammonium persulfate (APS), dimethyl sulfoxide (DMSO), methanol (MeOH), and normal melting point agarose were obtained from Roth (Karlsruhe, Germany). Acetylated bovine serum albumin (BSA), phenylmethanesulfonyl fluoride (PMSF), aprotinin, leupeptin, pepstatin, sodium fluoride (NaF), sodium orthovanadate (Na_3_VO_4_), dithiothreitol (DTT), APC, bicinchoninic acid (BCA) solution, formamide, blue dextran, Triton X-100, low melting point agarose, 1,4-Diazabicyclo[2.2.2]octane, potassium chloride (KCl), and potassium phosphate (KH_2_PO_4_) were obtained from Sigma/Merck (Darmstadt, Germany).

### 4.3. Incision Activity Assay Principles

This non-radioactive BER incision activity assay represents an advanced protocol based on the work of Hamann et al. [12]. We refined the scope of the assay from in vitro application for cultured cells to in vivo application for murine liver tissue samples. In addition, we broadened the assay spectrum to three oligonucleotides with different DNA lesions. This simplified the protocol and accelerated the tissue extract preparation. Also, it offered the possibility to study BER repair incision activity towards three common DNA lesions from a single tissue extract. In general, BER incision activity is studied by incubating Cy5-labelled hairpin-structured oligonucleotides incorporating different DNA lesions with a liver tissue extract that contains the damage incising repair enzymes. Hairpin-structure represents the more stable configuration compared to the frequently used duplex oligonucleotides [12]. The DNA lesions that proved successful incorporated into the oligonucleotides are the oxidative base modifications 8-oxodG and 5-OHdU [62], and an AP site analogue. Thus, repair enzyme-mediated processing of the DNA lesion leads to formation of a shorter, still fluorophore-labelled, oligonucleotide. Via a denaturing polyacrylamide gel electrophoresis (PAGE) the incised oligonucleotides were separated from the intact oligonucleotides, and subsequently quantified by fluorescence detection. 

### 4.4. Oligonucleotide Design

Design of oligonucleotides was based on a previously suggested base sequence harbouring the DNA lesion 8-oxodG [12]. In addition, this base sequence was shown to be suitable for investigation of incision activity towards an AP site analogue (tetrahydrofuran (THF) derivate) [57]. THF is commonly applied to mimic an AP site in oligonucleotides [63]. Further, we aimed at investigating another oxidative base modification: 5-OHdU. To include 5-OHdU into the assay, a previously described base sequence of a 5-OHdU containing oligonucleotide was chosen as a point of reference regarding arrangement of the bases surrounding 5-OHdU [24]. By merging oligonucleotide base sequences, we created a new oligonucleotide with hairpin-feature and 5-OHdU as DNA damage. Examination of hybridization efficiency and storage stability showed satisfying stability of all hybridized oligonucleotides. Fluorescence-dye labelled oligonucleotides, containing either a DNA lesion or the respective intact DNA base, were obtained from Eurogentec (Seraing, Belgium). Regarding 8-oxodG and the AP site analogue, oligonucleotides consisted of the base sequence

5’-CAATAATAACACGCXCGACCAGTCCTGCTTTTGCAGGACTGGTCGCGCGTGTTATTATTG-Cy5-3’ (X = 8-oxodG, AP site analogue). 

A further oligonucleotide with the identical base sequence, but with a G instead of a DNA lesion, was used as damage-free negative control. For 5-OHdU and the respective damage-free oligonucleotide the base sequence was

5’-CAATAATAACTCGTXACTTCAGTCCTGCTTTTGCAGGACTGAAGTGACGACTTATTATTG-Cy5-3’ (X = 5-OHdU or C). 

Prior to use, oligonucleotides were hybridized to form a double-stranded hairpin structure by heating to 90 °C for 15 min followed by slowly cooling down to 30 °C in 100 mM Tris-HCl (pH 8), 10 mM EDTA, and 50 mM NaCl. Hybridized oligonucleotides were stored at −20 °C until use. For all oligonucleotides, hybridization efficiency, and storage stability were monitored by native PAGE. 

### 4.5. Preparation of Tissue Extracts for Incision Activity

For preparation of tissue extracts, 1 mL of ice-cold extraction buffer (50 mM Tris-HCl (pH 7.1), 250 mM NaCl, 1 mM EDTA, 0.5 mM DTT, 20% glycerol, 0.5 mM PMSF, 10 µg/mL aprotinin, 5 µg/mL leupeptin, 1 µg/mL pepstatin, 50 mM NaF, 1mM Na_3_VO_4_, 1.5 µM APC (1.5 mM in DMSO)) was added to 50 mg of murine liver tissue. 30 zirconia beads (biolab, Bebensee, Germany) were added and the tissue was disrupted using a bead ruptor 12 (biolab, Bebensee, Germany). Tissue debris and beads were removed by centrifugation at 10,000 *g* and 4°C for 20 min. The supernatant was collected and, for purification, centrifuged again. The protein content of the tissue extracts was determined via the BCA assay and the protein solutions were stored at −80 °C. 

### 4.6. Incision Activity

Reaction buffer, tissue extract, and hybridised oligonucleotide were mixed to a final reaction volume of 10 µL containing 50 mM Tris-HCl (pH 7.1), 50 mM NaCl, 0.1 µg/µL acetylated BSA, and 50 fmol of oligonucleotide. After one-hour reaction time at 37 °C, the reaction was stopped by adding 10 µL of denaturation buffer (90% formamide, 1 mM EDTA (pH 8), 0.5% blue dextran), and heating to 95 °C for 5 min. Additionally, for each determination an oligonucleotide blank and a negative control consisting of the respective damage-free oligonucleotide incubated with tissue extract were included in the assay. Then, a denaturing PAGE (20% denaturing polyacrylamide gel (7 M urea, 89 mM Tris-borate, 2 mM EDTA (pH 8)), 2.5 h, 15 W per gel) followed to separate intact and incised oligonucleotides. For each animal, one tissue extract was prepared and one technical replicate was analysed for incision activity towards each of the three oligonucleotides. Detection and quantification of incision were conducted using a Chemidoc MP imaging system and the appropriate software Image Lab (Biorad, München, Germany). 

### 4.7. Analysis of PAR Levels

We conducted the sample preparation according to a previously published [37,64] and adapted [65] method, but with an optimized application for murine liver tissue. To extract PAR, 1 mL ice-cold trichloroacetic acid (TCA) (20%, w/v) was added to 10–20 mg of liver tissue. Tissue was disrupted using a tissue ruptor (Qiagen, Hilden, Germany) and subsequently centrifuged at 13,000 *g* and 4 °C for 15 min. The resulting pellet was washed twice with ice-cold 70% ethanol (EtOH) and air-dried at 37 °C. To detach protein-bound PAR, 600 µL 0.5 M potassium hydroxide (KOH) were added followed by thorough resuspension and incubation for 70 min at 37 °C. Tissue debris was separated by centrifugation at 13,000 *g* for 30 min and the supernatant was neutralized with 4.8 M 3-(N-morpholino)propanesulfonic acid (MOPS). 30 µL aliquots were prepared for determination of DNA concentration to normalise results. DNA concentration was determined applying a previously described method based on the use of Hoechst [65]. 

Further sample treatment included the addition of ^13^C,^15^N-labelled PAR as internal standard, digestion of nucleic acids, enrichment, and digestion of PAR to its monomeric units. Last, PAR quantification was conducted via LC-MS/MS as described in detail by Neumann and colleagues [65]. For each animal, one tissue sample was prepared for PAR measurement and one technical replicate was measured via LC-MS/MS.

### 4.8. Quantification of DNA Damage Levels

Cells were isolated mechanically by pressing 40 mg of snap frozen liver tissue through a cell strainer with 70 µm mesh size moving a plunger up and down for several times [66]. Isolated cells were flushed with 2 mL Merchant’s Medium (0.14 M NaCl, 1.47 mM KH_2_PO_4_, 2.7 mM KCl, 8.1 mM EDTA, pH 7.4) into a reaction tube below the strainer. Afterwards, the cell suspension was centrifuged at 100 *g* and 4 °C for 5 min and the supernatant was removed. 20 µL of cell suspension containing 2 × 10^6^ cells/mL were mixed with 180 µL of 0.5% low melting point agarose. 45 µL of the mixture were placed on a with 1.5% of normal melting point agarose pre-coated slide, covered with a cover glass and kept at 4 °C to solidify. After cover glass removal, slides were kept in a lysis solution (0.1% Triton X-100, 10% DMSO, and 89.9% lysis buffer containing 10 mM Tris, 2.5 M NaCl, and 100 mM Na_2_EDTA, pH 10) for 1 h at 4°C. After lysis, the slides were placed into an electrophoresis chamber filled with electrophoresis buffer (300 mM NaOH, 1 mM EDTA, pH 13) to allow the DNA unwinding at 4 °C. After 20 min of unwinding, electrophoresis was performed for 20 min at 25 V, 300 mA, and 4 °C. The slides were neutralized by immersion with 0.4 M Tris buffer (pH 7.5) for 5 min and were fixed for 5 min in ice-cold MeOH. Slides were stained with 20 µL of Gel red Nuclein Acid Gel Stain (10,000 × in H_2_O Biotium, Hayward, CA, USA) prior to microscopy with a florescence microscope (Leica DM 2000 LED, Leica Microsystems GmbH, Wetzlar, Germany). For each treatment, 200 randomly selected cells (100 per technical replicate slide) for each treatment were analysed by the semi-automated image analysis software (Comet IV, Perceptive Instruments, Stone, UK). The mean of the medians of 100 comets per technical replicate was calculated for each animal [39]. The percentage of DNA in tail was used to quantify the DNA damage. For each animal, cells were isolated from one tissue sample and two technical replicates were analysed for DNA damage. To determine the day-to-day-variation and electrophoresis efficiency, human liver carcinoma (HepG2) cells were used as negative and MMS-treated HepG2 cells as positive control. Further information on storage stability is provided in Figure S2. 

### 4.9. Statistical Analysis

Using GraphPad Prism 6 (version 6.01 for Windows, GraphPad Software, La Jolla, CA, USA), we tested for normal distribution using the Kolmogorov-Smirnov test. Because of the normal distribution of the data, two-tailed unpaired student’s *t*-tests were performed for group-wise comparisons between the two age-groups and the two sex-groups. Next, exploratory correlation analysis applying Spearman’s correlation was performed to assess the relationship between the analysed endpoints 8-oxodG, 5-OHdU, and AP site analogue incision activity, PARylation levels, and DNA damage levels. *p* < 0.05 was regarded as statistically significant. 

## Supplementary Materials

Supplementary materials can be found at https://www.mdpi.com/1422-0067/21/18/6600/s1.

## Abbreviations

5-OHdU	5-hydroxy-2’deoxyuracil
8-oxodG	8-oxo-2’-deoxyguanosine
AP	apurinic/apyrimidinic
APC	aphidicolin
APE	AP endonuclease
APS	ammonium persulfate
BCA	bicinchoninic acid
BER	base excision repair
BSA	bovine serum albumin
CCD	charge-coupled device
DDR	DNA damage response
DMSO	dimethyl sulfoxide
DTT	dithiothreitol
EtOH	ethanol
HCl	hydrochloric acid
HEPES	4-(2-hydroxyethyl)piperazine-1-ethanesulfonic acid
KCl	potassium chloride
KH_2_PO_4_	potassium dihydrogen phosphate
KOH	potassium hydroxide
LC-MS/MS	liquid chromatography tandem mass spectrometry
MeOH	methanol
MOPS	3-(N-morpholino)propanesulfonic acid
NaCl	sodium chloride
Na_2_EDTA	disodium ethylenediaminetetraacetate
NaF	sodium fluoride
NaOH	sodium hydroxide
Na_3_VO_4_	sodium orthovanadate
NEIL	Nei-like DNA glycosylase
NTH	Nth-like DNA glycosylase
OGG1	8-oxoguanine DNA glycosylase 1
PAGE	polyacrylamide gel electrophoresis
PAR	poly(ADP-ribose)
PARP(s)	poly(ADP-ribose) polymerase(s)
PARylation	poly(ADP-ribosyl)ation
PMSF	phenylmethanesulfonyl fluoride
ROS	reactive oxygen species
TCA	trichloroacetic acid
TEMED	N,N,N′,N′-Tetramethylethylenediamine
THF	tetrahydrofuran
Tris	2-amino-2-(hydroxymethyl)-1,3-propanediol
