# Supplementary material for: A Multi-Endpoint Approach to Base Excision Repair Incision Activity Augmented by PARylation and DNA Damage Levels in Mice: Impact of Sex and Age
Source: Int J Mol Sci. 2020 Sep 9;21(18):6600. doi: 10.3390/ijms21186600 (PMC7555950; doi:10.3390/ijms21186600)
Supplement: Supplementary file 1 [file ijms-21-06600-s001.pdf]

A Multi-Endpoint Approach to Base Excision Repair Incision Activity Augmented by PARylation and DNA Damage Levels in Mice: Impact of Sex and Age

-Supplementary Material-

Nicola Winkelbeiner [1,2,#], Viktoria K. Wandt [1,2,#], Franziska Ebert [1,2], Kristina Lossow [2,3,4], Ezgi E. Bankoglu [5], Maximilian Martin [1], Aswin Mangerich [6], Helga Stopper [5], Julia Bornhorst [2,7], Anna P. Kipp [2,3] and Tanja Schwerdtle [1,2,8,*]

[1]Department of Food Chemistry, Institute of Nutritional Science, University of Potsdam, Arthur-Scheunert-Allee 114-116, 14558 Nuthetal, Germany; tanja.schwerdtle@uni-potsdam.de

[2]TraceAge – DFG Research Unit on Interactions of Essential Trace Elements in Healthy and Diseased Elderly (FOR 2558), Berlin-Potsdam-Jena-Wuppertal, Germany

[3]Department of Molecular Nutritional Physiology, Institute of Nutritional Sciences, Friedrich Schiller University Jena, Dornburger Straße 24, 07743 Jena, Germany; anna.kipp@uni-jena.de

[4]German Institute of Human Nutrition, Arthur-Scheunert-Allee 114-116, 14558 Nuthetal, Germany; kristina.lossow@uni-jena.de

[5]Institute of Pharmacology and Toxicology, University of Würzburg, Versbacher Straße 9, 97078 Würzburg, Germany; stopper@toxi.uni-wuerzburg.de

[6]Department of Biology, Molecular Toxicology Group, University of Konstanz, Universitätsstraße 10, 78457 Konstanz, Germany; aswin.mangerich@uni-konstanz.de

[7]Food Chemistry, Faculty of Mathematics and Natural Sciences, University of Wuppertal, Gaußstraße 20, 42119 Wuppertal, Germany; bornhorst@uni-wuppertal.de

[8]German Federal Institute for Risk Assessment (BfR), Max-Dohrn-Strasse 8-10, 10589 Berlin, Germany

[*]Correspondence: tanja.schwerdtle@uni-potsdam.de; Tel.: +49-33200-885528

[#]These authors contributed equally to this work.

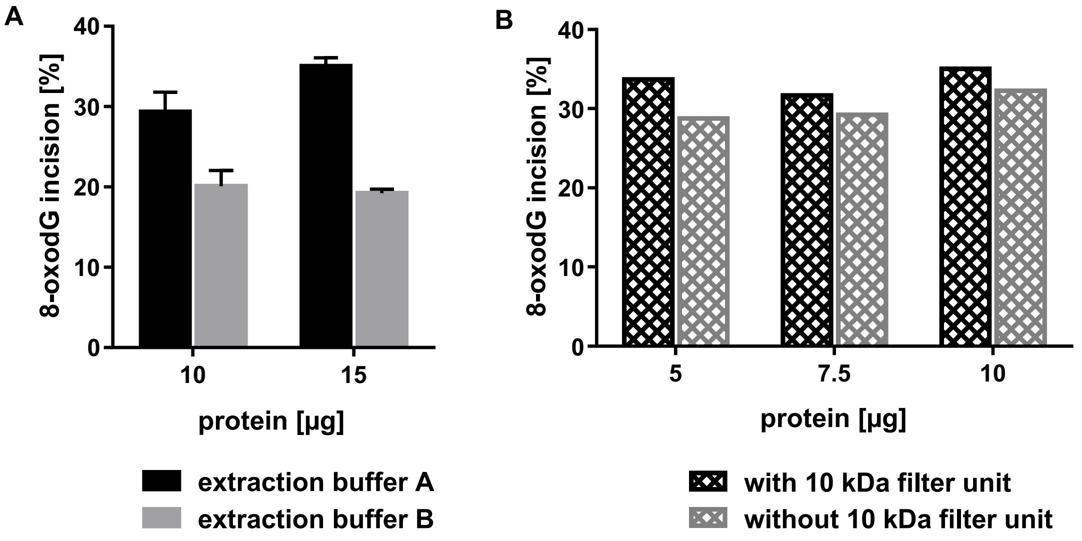

Figure S1: Optimisation of tissue extract preparation. (A) Mean incision activity + SEM of different amounts of murine liver extract protein towards the 8-oxodG containing oligonucleotide. Tissue extracts of two mice were prepared in two different extraction buffers. Composition of extraction buffer A is based on the *in vitro* protocol, whereas extraction buffer B composition is derived from a comet-based repair assay. (B) Incision activity of different amounts of murine liver extract protein prepared form one mouse towards the 8-oxodG containing oligonucleotide, prepared with or without the use of filter units.

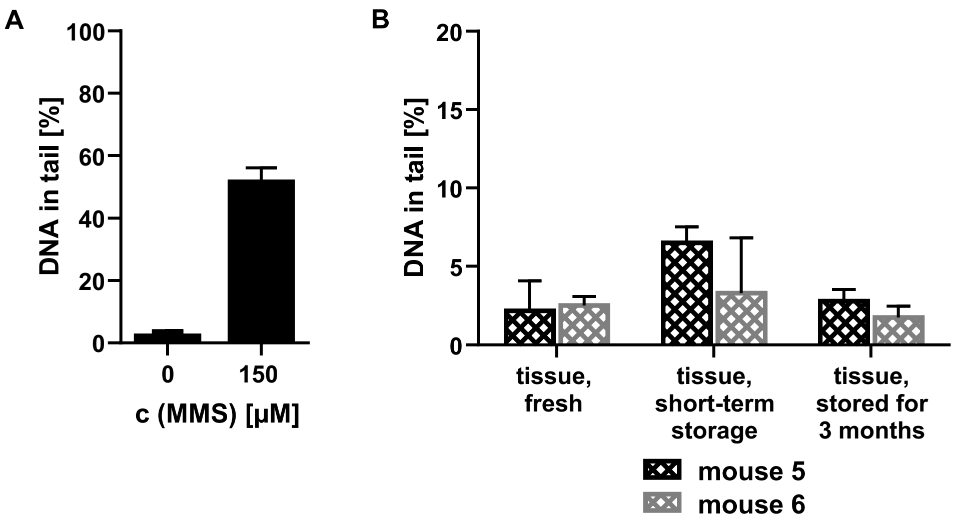

Figure S2: (A) DNA damage levels of assay control. Frozen HepG2 cells were used as negative and 4 h with methyl methanesulfonate (MMS) treated cells as positive assay control. Samples were frozen in 90% fetal calf serum and 10% dimethyl sulfoxide and stored at − 80°C until reconditioning. To examine day-to-day variation and electrophoresis efficiency samples were included in every performed alkaline comet assay. Shown are mean values of 3 experiments + SD. (B) Comparison of DNA strand break levels in fresh and frozen liver tissue. DNA strand break levels were analysed via alkaline comet assay in fresh and snap frozen (immediately after dissection) liver tissue stored at −80°C for short-term (max. 5 days) or long-term (4 months). Shown are mean values of two hundred randomly selected cells (100 per technical replicate) + SD of two different untreated mice.

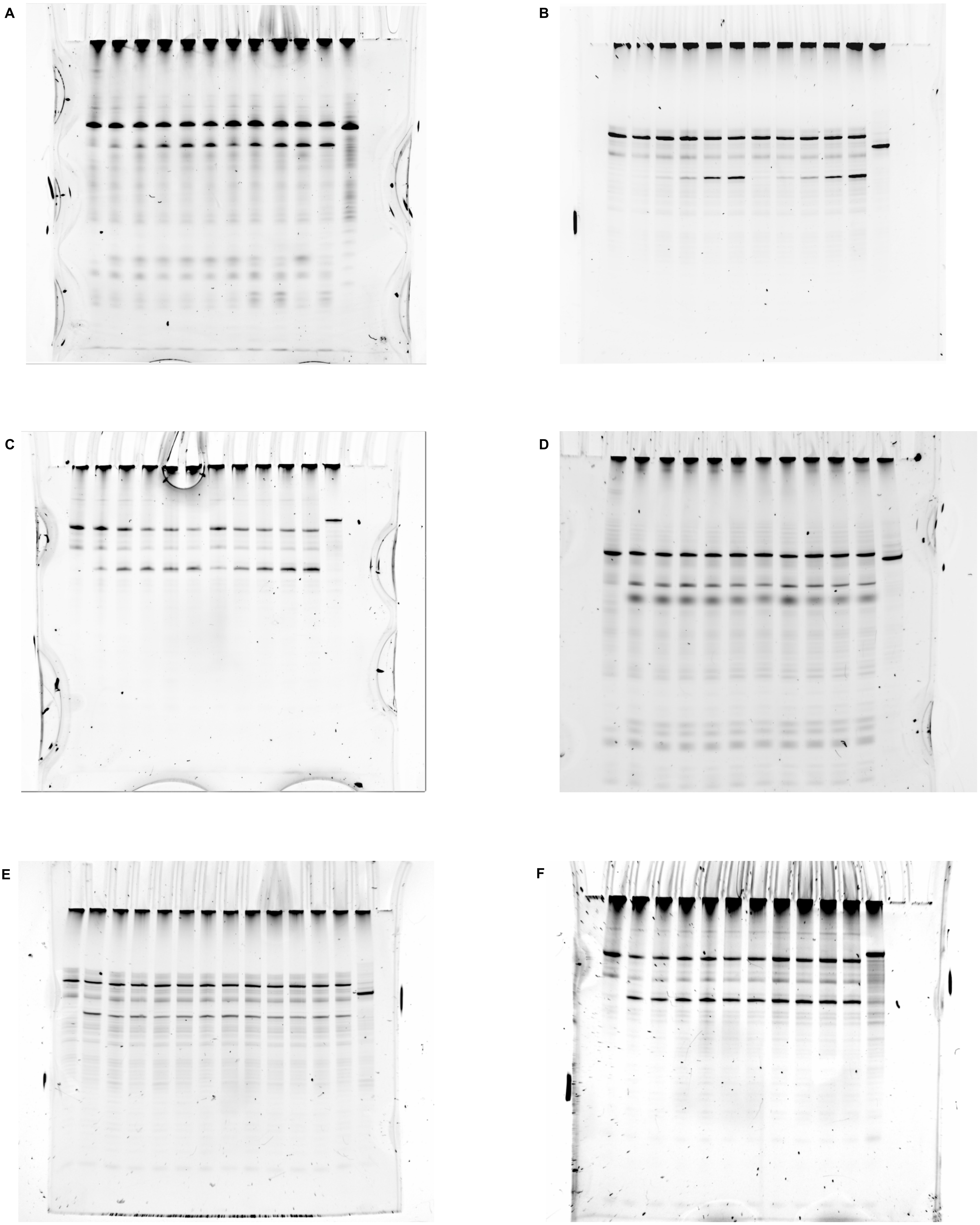

Figure S3: Full-length PAGE gels of BER incision activity analysis. Shown are full-length polyacrylamide gels with Cy5-labelled oligonucleotide bands referring to (A) Figure 1B, (B) Figure 1C, (C) Figure 1D, (D) Figure 3G, (E) Figure 3H, and (F) Figure 3I.
